# Supplementary material for: MYB and ELF3 differentially modulate labor-inducing gene expression in myometrial cells
Source: PLoS One. 2023 Jan 3;18(1):e0271081. doi: 10.1371/journal.pone.0271081 (PMC9810189; doi:10.1371/journal.pone.0271081)
Supplement: S1 File — (DOCX) [file pone.0271081.s001.docx]

**Supporting Information for:** MYB and ELF3 differentially modulate labor-inducing gene expression in myometrial cells

Author List and Affiliations

Virlana M. Shchuka^1^, Nawrah Khader^1^, Anna Dorogin^2,3^, Oksana Shynlova^2, 3^, and Jennifer A. Mitchell^1^

1. Department of Cell and Systems Biology, University of Toronto, Toronto, ON, Canada.

2. Lunenfeld Tanenbaum Research Institute, Sinai Health System, Toronto, ON, Canada.

3. Department of Obstetrics and Gynaecology, University of Toronto, ON, Canada.

Current address: Department of Cell and Systems Biology, University of Toronto, Toronto, Canada

Corresponding authors:

virlana.shchuka@mail.utoronto.ca, ja.mitchell@utoronto.ca, shynlova@lunenfeld.ca

**Fig S1.** **Quiescent and term laboring myometrial transcriptomes in Bl6 mice exhibit time point-distinguishing *Myb* and *Elf3* expression profiles.** **(A)** Schematic of gestational time points of interest for RNA collection from Bl6 mouse myometrial tissues. Gestational days (d) at which tissues were collected marked by triangles (red), with indication of term non-laboring (TNIL), term labor (TL) and postpartum (PP) time points alongside key gestational stages (*italicized*). **(B)** Expression levels (±S.D.) of transcription factor-encoding genes at d15, d18.75, d19.5, and d20 time points, as determined by RT-qPCR. Groups determined by one-way ANOVA to be significantly different (p < 0.05) from one another are labeled with different letters; to indicate p > 0.05, groups are labeled with the same letter.

**Fig S2. Cells transfected with transcription factor-encoding constructs express said factors at the protein level.** Protein levels of factor encoded by designated construct, with pCDNA3.1 (pcDNA) empty vector used as negative control (CTL).

**Table S1. List of primers used in mouse labor-upregulated TF expression quantification experiments.**

| **TF Transcript Target** | **Forward Primer 5’->3’** | **Reverse Primer 5’->3’** | **Size (bp)** | **Refseq Accession Numbers** |
| --- | --- | --- | --- | --- |
| Myb | CGGCGATGTGGTAATAGGGATAT | CACGAGCTTCCAGAAGAACAATC | 119 | [NM_001198914.1](https://www.ncbi.nlm.nih.gov/entrez/viewer.fcgi?db=nucleotide&id=312032463) |
| Elf3 | GCTCAGCTTCTCATAGGTCATGT | GAGGGTGTGTTCAAGTTTCTTCG | 93 | [NM_001163131.1](https://www.ncbi.nlm.nih.gov/entrez/viewer.fcgi?db=nucleotide&id=253683410) |
| H1f0 | GGCCAAGGCTTCCAAGAAGT | CCACCTTGTAGTGGCTCTTGATA | 137 | [NM_008197.3](https://www.ncbi.nlm.nih.gov/entrez/viewer.fcgi?db=nucleotide&id=85838505) |
| Tbp | CTCAGTTACAGGTGGCAGCA | ACCAACAATCACCAACAGCA | 187 | [NM_013684.3](https://www.ncbi.nlm.nih.gov/entrez/viewer.fcgi?db=nucleotide&id=172073170) |

**Table S2. List of primers used in human labor-upregulated TF expression quantification experiments.**

| **TF Transcript Target** | **Forward Primer 5’->3’** | **Reverse Primer 5’->3’** | **Size (bp)** | **Refseq Accession Numbers** |
| --- | --- | --- | --- | --- |
| MYB | GAATTCTACAATGCGTCGGAAGG | ATAGTCGTTGTTAACAGTGGGCT | 178 | [NM_001130173.2](https://www.ncbi.nlm.nih.gov/entrez/viewer.fcgi?db=nucleotide&id=1519242645) |
| ELF3 | CCTCATGAAGTGGGAGAATCGG | CTCAGCTTCTCGTAGGTCATGTT | 117 | [NM_004433.5](https://www.ncbi.nlm.nih.gov/entrez/viewer.fcgi?db=nucleotide&id=1519314459) |
| MAPK1 | ATTTTAACCCTCTGAGGATCTGG | GTACAGGACCTCATGGAAACAGA | 106 | [NM_002745.5](https://www.ncbi.nlm.nih.gov/entrez/viewer.fcgi?db=nucleotide&id=1777376004) |

**Table S3. List of primers used to clone labor-associated gene promoters into target reporter vectors for luciferase assays.** Lowercase letters represent additional portion of *Gja1* coding region before the first nucleotide in the codon encoding the first Met residue (*Gja1*) or the overhang portion of the primer corresponding to the sequence in the target vector backbone. Refseq accession numbers obtained from portion of primer sequences that does not correspond to overhang.

| **Promoter** | **Forward Primer 5’->3’** | **Reverse Primer 5’->3’** | **Size (bp)** | **Refseq Accession Numbers** |
| --- | --- | --- | --- | --- |
| *Gja1* | TCTCCTGAAGGAATGACCCATCCA | gtctgggcacctcTCTTTCACTTAATGAAAGTGAAGCC | 503 | [NC_000076.7](https://www.ncbi.nlm.nih.gov/nucleotide/1877089959?from=34518206&to=56253614&report=gbwithparts) |
| *Gja1* (cloning from transitional vector) | gaggatatcaagatctTCTCCTGAAGGAATGACCCATCCA | ttggcatcttccatggGTCTGGGCACCTCTCTTTCACT | n/a | n/a |
| *Fos* | gaggatatcaagatctACTTATTTACAATCCTTCACTTGCT | ttggcatcttccatggGGTCGAAGTTTGGGGAAAGCC | 901 | [NC_000078.7](https://www.ncbi.nlm.nih.gov/nucleotide/1877089957?from=85519913&to=85520813&report=gbwithparts) |
