# Supplementary figures and images for: MYB and ELF3 differentially modulate labor-inducing gene expression in myometrial cells
Source: PLoS One. 2023 Jan 3;18(1):e0271081. doi: 10.1371/journal.pone.0271081 (PMC9810189; doi:10.1371/journal.pone.0271081)

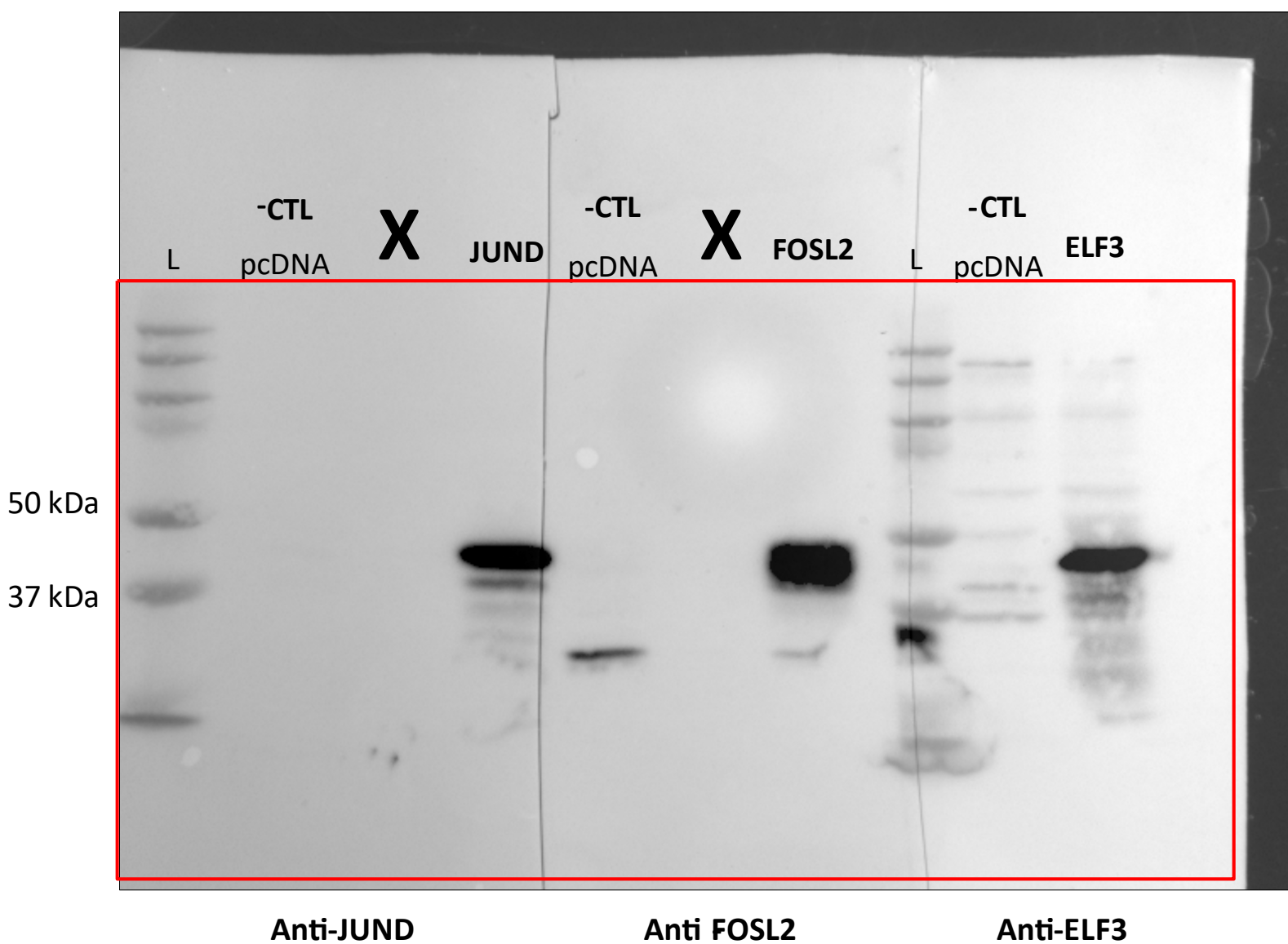

**\*Figure used to generate SFig2 (upper panel)**

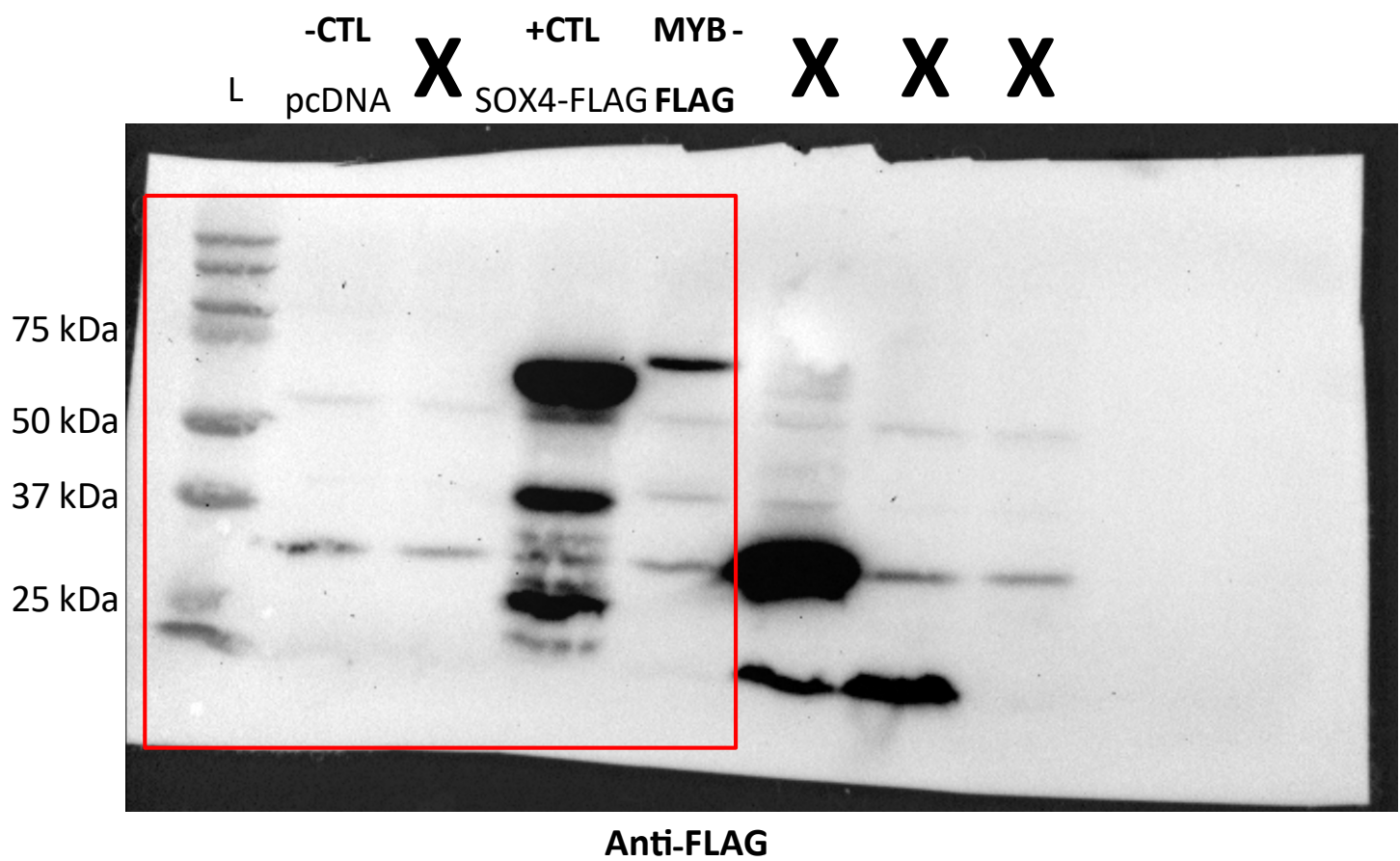

**\*Figure used to generate SFig2 (lower panel)**

Supplement: S1 Raw images — (PDF) [file pone.0271081.s002.pdf]
